# Basics of Sustainable Diets and Tools for Assessing Dietary Sustainability: A Primer for Researchers and Policy Actors

**DOI:** 10.3390/healthcare10091668

**Published:** 2022-08-31

**Authors:** Ioanna Alexandropoulou, Dimitrios G. Goulis, Theodora Merou, Tonia Vassilakou, Dimitrios P. Bogdanos, Maria G. Grammatikopoulou

**Affiliations:** 1 Department of Nutritional Sciences & Dietetics, Faculty of Health Sciences, International Hellenic University, Alexander Campus, GR-57400 Thessaloniki, Greece; 2 Unit of Reproductive Endocrinology, 1st Department of Obstetrics and Gynecology, Medical School, Aristotle University of Thessaloniki, 76 Agiou Pavlou Street, GR-56429 Thessaloniki, Greece; 3 Department of Forest and Natural Environment Sciences, International Hellenic University, GR-66100 Drama, Greece; 4 Department of Public Health Policy, School of Public Health, University of West Attica, 196 Alexandras Avenue, GR-11521 Athens, Greece; 5 Department of Rheumatology and Clinical Immunology, Faculty of Medicine, School of Health Sciences, University of Thessaly, Biopolis, GR-41110 Larissa, Greece

**Keywords:** environment, plant-based diet, resilience, one health, ecological footprint, water footprint, greenhouse gas, land occupation, sustainable diet index, Sustainable-HEalthy-Diet (SHED) index, food packaging, business as usual

## Abstract

Climate change can have economic consequences, affecting the nutritional intake of populations and increasing food insecurity, as it negatively affects diet quality parameters. One way to mitigate these consequences is to change the way we produce and consume our food. A healthy and sustainable diet aims to promote and achieve the physical, mental, and social well-being of the populations at all life stages, while protecting and safeguarding the resources of the planet and preserving biodiversity. Over the past few years, several indexes have been developed to evaluate dietary sustainability, most of them based on the EAT-*Lancet* reference diet. The present review explains the problems that arise in human nutrition as a result of climate change and presents currently available diet sustainability indexes and their applications and limitations, in an effort to aid researchers and policy actors in identifying aspects that need improvement in the development of relevant indexes. Overall, great heterogeneity exists among the indicators included in the available indexes and their methodology. Furthermore, many indexes do not adequately account for the diets’ environmental impact, whereas others fall short in the economic impact domain, or the ethical aspects of sustainability. The present review reveals that the design of one environmentally friendly diet that is appropriate for all cultures, populations, patients, and geographic locations is a difficult task. For this, the development of sustainable and healthy diet recommendations that are region-specific and culturally specific, and simultaneously encompass all aspects of sustainability, is required.

## 1. Introduction

Despite the numerous technological advantages and the evolution of science, the agricultural and fishery sectors still fail to fulfill the dietary needs of the global population [1]. In addition, climate change has economic consequences and increases the threat of food insecurity, as it negatively affects agricultural and livestock production, and also fisheries [2,3,4]. With regard to human nutrition, climate change impacts food quality parameters, including protein, micronutrients, and vitamin content, mainly in basic crops [5]. For proteins in particular, the projected estimated reduction in some basic grains, such as rice and barley, can have an important impact on population health [5,6]. On the other end of the spectrum, human nutrition also impacts climate change by increasing greenhouse gas emissions (GHGe, including methane, CO_2_, and N_2_O), overconsumption of water resources, and extensive land use [7]. It was found that one-quarter of GHGe are released due to agricultural activity, with animal husbandry accounting for the largest percentage [8]. In addition, studies revealed that dietary patterns with high animal food content are releasing greater GHGe. The increase in GHGe, mainly due to human activity, leads to an increase in the planet’s temperature, resulting in the observed climate change and the effects it brings [9].

**Figure 1:**
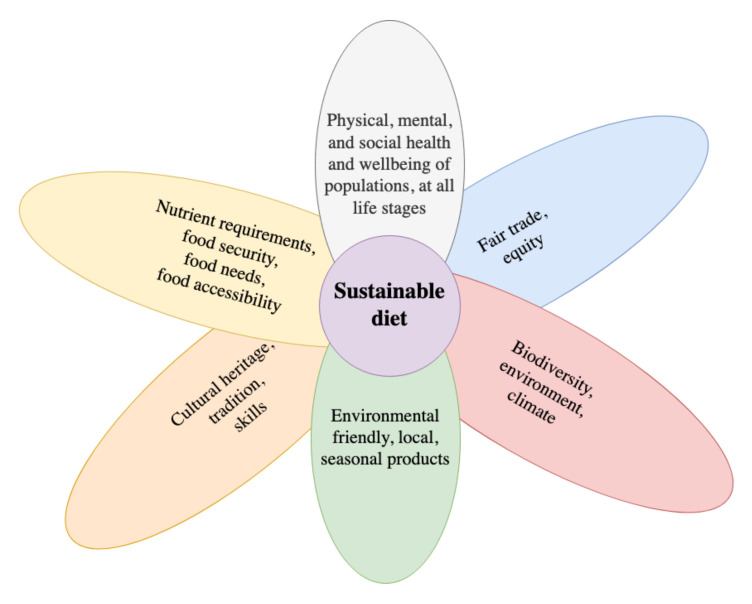
Components of sustainable diets according to the FAO [11].

Counterbalancing the results of climate change on our diet requires changing the way we produce and consume food. The development of sustainable food standards is a key objective of the United Nations (UN), aiming to achieve the Sustainable Development Goals (SDGs) [10]. Although we currently lack a unanimous definition of what a sustainable diet is, according to the Food and Agriculture Organization (FAO) (Figure 1) [11], it aims to achieve and maintain the physical, mental, and social health and well-being of the populations at all life stages, while protecting and safeguarding the resources of the planet and preserving the biodiversity [12]. In this context, a sustainable diet is also an accessible one, ensuring equity while based on fair trade principles, encompassing eco-friendly, local, and seasonal foods that are covering the nutritional needs of the people, limiting food insecurity [11].

Since the EAT-*Lancet* Commission on healthy diets from sustainable food systems was published [7], consumers’ concerns for the critical role of food systems were heightened, propelling research interest in achieving and quantifying a sustainable diet. Clearly, a single agenda integrating global health and environmental sustainability would not be adequate in achieving the SDGs. Hence, various tools and indexes have been designed and proposed, aiming to assess dietary sustainability using different components and food groups. A variety of indicators can be used to evaluate the impact of certain food groups on the environment, with GHGe being the most used in research [13]. Other indicators include pesticides, land and blue water use, biodiversity, and eutrophication (i.e., the increase in the supply rate of organic matter to an ecosystem) [14].

There is a need to recognize the impact of human nutrition on the environment and how changes in the way we eat will contribute to a more sustainable environment. There is also a need to measure how sustainable the population’s diet is and calculate its environmental impact. Thus, it is necessary to describe the current indexes, their advantages and disadvantages, and how to use them, in order to aid researchers and policy actors in identifying aspects that need improvement in the development of novel indexes.

For this reason, the aims of the present review are (a) to understand the problems that arise in human nutrition due to climate change and present sustainable food systems as one of solutions to the problem, and (b) to showcase the existing indexes/tools for evaluating sustainable nutrition, as well as the advantages and disadvantages that each one displays.

## 2. Nutrition and Climate Change

Climate change poses a threat to food security, mainly due to its negative impacts on agricultural productivity. In the past few decades, research has also focused on how climate change affects main crops’ quality parameters [2,5]. Recent studies suggest that the change in the climate will lead to modifications in the nutrient content of basic staple foods consumed in our daily diet, including rice, barley, flour, legumes, etc. [15,16,17]. In some cases, these modifications concern reductions in the content of basic nutrients, such as proteins, but with regard to the micronutrient content of foods, including iron (Fe) and zinc (Zn) levels. The content of staple crops with respect to these micronutrients is an important determinant in estimating the dietary intake of micronutrients in a population [15,16]. Nutrient deficiencies involving Fe and Zn constitute major global public health issues, particularly in areas where people base their dietary intake on these crops, such as women and children residing in sub-Saharan Africa [14]. The main parameters of climate change affecting food nutrient composition include the increasing ambient temperature, followed by drought and the subsequent increases in atmospheric CO_2_ concentration.

### 2.1. Effects of Drought

Drought reduces the concentration of nutrients in plants such as legumes, cereals, and grasses. In particular, it affects the cultivation of beans and *Hordeum vulgare* (barley), *Zeamays* (Zea), and ryegrass [15]. Common beans are the most important legumes supporting food security and human nutrition globally. In particular, they provide almost 15% of daily calories and 36% of daily protein in many African and South American countries [18]. For smallholder farmers in sub-Saharan Africa, beans are an important crop and a key nutritional component in the diets of poor rural communities. Inevitably, such small farming communities are more vulnerable to the negative effects of climate change [19,20]. For common beans, it is not known whether drought-related crop reductions will also lead to changes in nutritional quality under future drought scenarios [21]. A modeling study investigating the impact of changes in heat and rainfall frequency in southeastern Africa [15] predicted that by the year 2050, common beans will become unsuitable for cultivation in the vast majority of the current bean-growing areas. Thus, the transfer and relocation of crops to new areas should be performed with careful selection of suitable varieties for each new geographical area [15].

Drought also reduces approximately 50% the nitrogen (N) and phosphorus (P) concentration in the susceptible barley and Zea plants and in the drought-resistant *Andropogon gerardii* (big bluestem). This is due to the decrease in the level of nutrient uptake proteins or transporters in plant roots, such as the NRT1 protein for the uptake of nitrate (NO_3_), the ammonium transporter 1 (AMT1) for ammonium (NH_4_), and the PHT1 transporter for P [17]. The negative effects of drought have also been detected on the mRNA levels of nutrient uptake proteins, including the Zn transporter in maize, the silicon (Si) transporter in rice, and the NO_3_ and sulfate (SO_4_) transporters in grapes [22]. In parallel, heat stress also induces negative effects on the antioxidant enzyme content of *Zea mays* [23]. In contrast, up-regulation of the NRT1, AMT1, and PHT1 proteins per unit of total root protein during drought was observed in a recent study, associated with increases in the mRNA levels of these proteins in maize [24]. However, since nutrient uptake proteins can be post-transcriptionally regulated, it is important to assess the levels of these components in order to understand how exposure to drought affects their expression and uptake rate [25].

### 2.2. Increase in CO_2_ Concentration

Over the next 30–80 years, the rate of increase in atmospheric CO_2_ is likely to exceed 550 ppm. According to the literature, increased levels of CO_2_ in the atmosphere will affect plant growth and suppress Zn, Fe, and protein levels in staple crops. In a recent study conducted by Smith and Myers [16], 41 cultivars of six food crops were grown over 10 years on three continents to determine the effect of elevated CO_2_ levels on their quality. The results revealed reduced levels of Fe and Zn in all crops. In another study, protein levels were also decreased in grains, but legumes were not as affected, probably due to the general ability of legumes to extract and assimilate more N than other grains under increased CO_2_ exposure in order to maintain the C:N ratio (25). The levels of some vitamins have been reported to change. For example, Zhu and associates [5] found that in rice, the level of vitamin E increased, while other vitamins (vitamins B_1_, B_2_, B_5_, and B_9_) were reduced.

Another research group [26] studied the effects of increased CO_2_ on plant growth in greenhouses, or indoors. The results revealed that in artificial environments, the changes, particularly the increase in CO_2_, can negatively affect the concentration of nutrients in some plants [26]. C3 plants, such as rice and wheat, have less energy-efficient photosynthesis compared to C4 plants, such as maize [24]. This is due to the different photosynthesis mechanism in these plants. In C3 plants, the first product of photosynthesis is a sugar with three carbon atoms, while C4 plants produce sugar with four carbon atoms. C4s are very efficient at using CO_2_ and thus lose less water [27]. If a C3 plant grows in an environment abundant in CO_2_, then it will produce more carbohydrates, but it will concurrently reduce its concentration of other elements, including vitamins of the B complex. In particular, if wheat is grown in a CO_2_ concentration exceeding 546 ppm, then it will have approximately 6–13% less protein, 4–7% less Zn, and 5–8% less Fe [28].

In field experiments grown under elevated CO_2_ conditions, Meden and associates [6] observed a reduction in the protein content of rice, barley, potato, and wheat, ranging from 6.5% to 14%. The negative effect of high ambient CO_2_ concentration on the Zn, Fe, and protein content of plants was also shown in experiments involving different rice varieties in China and Japan [5]. In the same study, the effects on the levels of B vitamins were also investigated and it was observed that the reduction in the nutrient content of rice was even greater, ranging between 13% and 30%, with the greatest reduction in folic acid concentrations. On the other hand, an increase in vitamin E (α-tocopherol) levels was also observed [5].

### 2.3. Effects on Human Health

As already mentioned, increases in atmospheric CO_2_ content induce significant reductions in the protein content of plants such as rice, potatoes, wheat, and barley. Therefore, populations that depend on these plants to meet their dietary protein goals are expected to experience a reduction in their protein intake, exceeding 5% [6]. According to Medek [6], it has been estimated that by the year 2050, approximately 150 million people may be at risk of protein deficiency as a consequence of increased ambient CO_2_. Zhu and associates [5] used a food balance sheet developed by the FAO and gross domestic product (GDP) to estimate the risk of nutritional deficiencies for the 10 largest rice-consuming countries. Their results indicated that shortfalls were larger for countries with the lowest GDP [5]. Another study suggested that the countries at greater risk include Tajikistan, Bangladesh, Burundi, Liberia, the Occupied Palestinian Territories, Iraq, and Afghanistan, while in India, an additional 5% of the population is estimated to be at risk of developing protein deficiency due to the changes in rice content [6].

According to Bennett’s law, an increased income can provide alternative food sources for energy, and consequently protein, including meat, fish, and dairy, whereas low income is associated with more carbohydrate intake and reduced protein sources [29]. Therefore, in economically developed countries such as South Korea and Japan, the reduced protein in rice is not expected to alter the dietary intake of the populations. In contrast, in developing countries such as Guinea and Madagascar, dependence on rice persists. Hence, poorer countries are bound to be more affected by this nutritional deficit [5]. These predicted results are based on the Intergovernmental Panel on Climate Change’s climatic scenarios, which estimate that CO_2_ concentrations will reach 570 μmol mole^−1^ by the end of the century [30].

Based on these observations, Smith and Myers [31] estimated the public health implications of the deficiency of dietary B-complex vitamins in rice as a result of climate change. They also tried to quantify the risk of developing neural tube defects in babies born from mothers suffering from folate deficiency. Their results showed an increased risk for folate, thiamine, and riboflavin deficiencies in an additional 132, 67, and 40 million people, respectively. They used a statistical model in which the data came from estimates of nutrient stocks and corresponding requirements at a national level [31].

In parts of the world with a great prevalence of food insecurity, the reduced nutritional value of cheap and easily accessible diet seeds exacerbates the problem. It is estimated that 1.4 billion women and children under the age of five living in countries with high rates of anemia will lose an additional 4% of their dietary Fe intake. Iron deficiency can lead to stunted growth and reduced mental and physical abilities. In countries where manual labor is the main type of work, this can have a negative effect on income, and therefore further limit access to adequate and nutritious food [16]. Moreover, nutrient depletion projections suggest that by the year 2050, an additional 175 million people worldwide will be Zn-deficient [16]. As a result, geographic areas exhibiting less diet diversity such as India, sub-Saharan Africa, and parts of South Asia will be more affected by changes in the nutrient levels of staple crops [16].

### 2.4. Impact of Human Nutrition on Climate Change

Food is a basic human right and a healthy diet can contribute to our health and well-being. Distinct types of foods differ, not only in terms of their nutrient content, but also in the amount of land, water, energy, and GHGe required to produce them (ecological footprint) [32]. Today, the complex and global food production and distribution system is created to meet our ever-increasing nutritional needs, in terms of both quantity and taste. Nowadays, it is possible, for example, to fish in the Atlantic Ocean and consume the products within a few days on the European continent, as well as to supply European products all over the world.

Nonetheless, for many scientists, the food sector is considered an important effector of environmental change. It has been estimated that 30% of the GHGe are food-derived, while 70% of the drinking water is used for food production globally. The extensive use of land for crops has altered natural ecosystems, leading to the extinction of several species [7]. The rate of extinction and reduction in species population is greater than that of the Holocene era and is estimated at 1 species/million species/year. The amount in kilograms of insects decreased by 75% in the past three decades, while that of domestic birds decreased by 30% in one and a half decades [7]. Results from food life cycle assessment (LCA) studies reveal that the environmental impacts of food production are higher for animal products, and even greater for ruminants [33].

Total food demand is expected to increase by 70% by the year 2050, with a shift to animal-based foods including seafood, the consumption of which is expected to increase by 80%. It is believed that the global demand for beef may increase by 95% [9]. A consequence of the ever-higher consumption of animal products, and especially meat, is the increase in GHGe, which may reach 80% by the year 2050. The long-term overconsumption of meat can also affect the availability and prices of other food products [9]. This increasing consumption is mainly located in the urban areas of emerging economies in Asia, such as India and China [34]. In the U.S. and Europe, a decrease in beef consumption per capita was noticed since the 1970s, mainly due to public health concerns and rise in ready-to-go foods that give more alternatives. Projections indicate that beef consumption/capita on the European continent is expected to remain almost steady. Nevertheless, the consumed amount/person/day of animal products remains higher among high-income countries compared to low-income ones [9,35].

### 2.5. Sustainable Diets

The change in the way we produce and consume food is a key goal for the UN in order to achieve the SDGs. There are four SDGs for sustainable food systems and healthy eating standards: (a) SDG 2 aims to stop hunger, accomplish food security, enhance nutrition, and encourage sustainable agriculture; (b) SDG 3 aims to ensure healthy living and supporting well-being for everyone at any age; (c) SDG 12 aims to establish sustainability in producing and wasting systems; and (d) SDG 13 addresses the need for immediate actions to combat the effects of climate change [10].

The concept of a sustainable diet has been around for several years and links the environmental impact of our food choices. It includes meeting nutritional requirements with diets having a low environmental impact and respecting the biodiversity and cultural heritage of each region, and at the same time, it covers the parameters of accessibility and affordability. Studies have shown that these diets can reduce GHGe via the reduction in the intake of animal foods and their replacement with plant foods [36]. According to the FAO [11], a sustainable and healthy diet should take into account several factors. The most important of them are environmental (climate change, loss of biodiversity, land use, air and water pollution), food security issues (access to it, availability, stability of production), nutritional adequacy (enrichment with all necessary macro- and micro-nutrients), people’s socio-economic background, culture, and animal welfare issues [11].

These diets should be designed to be specific to each distinct environment, and therefore, there is no ideal dietary pattern for all. Our knowledge is growing about healthier eating habits with a lower environmental impact, but we should also include the socio-economic dimensions of sustainability in this context. We already know that the nutritional needs differ between sexes and age groups, but this is also happening between countries. There are different energy and dietary requirements for macro- and micronutrients between low- and high-income countries [37].

In addition, a review comparing the results of several studies relating different dietary patterns to the produced GHGe and the land use required for the production of the diets revealed that the least GHGe and land use requirements were observed in vegetarian and other “healthy” diets. Nevertheless, the substitution of red meat with poultry may induce a lower impact on global warming [38]. However, there is the risk that reducing animal foods will lead to a subsequent reduction in protein intake, so ideally, they should be replaced by alternative protein sources. In this context, researchers also focused on identifying alternative sources of protein with a smaller environmental footprint. MacDiarmid and Whybrow [36] reviewed relevant studies investigating (a) the use of already existing protein-rich plant foods, such as legumes; (b) alternative animal sources, such as edible insects; and (c) the development of novel foods, including lab-grown meat. Studies based on the LCA revealed that insect-based products had a lower environmental impact, including lower GHGe and land use, compared to animal products [39]. In contrast, in the case of lab-grown meat, the lower environmental impact compared to animal products is dependent on the production unit. Further studies are required to clarify the suitability of alternative protein sources [40].

The aforementioned issues associated with climate change and the projection trends indicate that the time for action and changing the food system is now. The lack of specific scientific targets for what a healthy and sustainable diet constitutes was apparent [7], leading to the development of specific expert groups aiming to solve the problem.

## 3. The EAT-*Lancet* Reference Diet

The EAT-*Lancet* Commission on Food, Planet, Health brought together 37 renowned scientists to evaluate if the future population can be fed on a healthy diet within planetary boundaries [41]. The Commission proposed the first targets for the consumption of a sustainable diet and the environmental boundaries regarding food production in order to meet the SDGs [42]. These targets formed the eponymous reference diet based on the universal diet that theoretically promotes human and environmental health simultaneously, under the “one health” principle [7]. It is based on eight food groups: whole grains, tubers and starchy vegetables (potatoes and cassava), vegetables, fruits, dairy foods, protein sources (meat, eggs, fish, legumes, nuts), added fats, and added sugars. The diet is mainly plant-based, with whole grains, vegetables, fruits, nuts, and legumes comprising the majority of the consumed foods. Meat and dairy are also included in the diet, but their target consumption is significantly less than that of plant-products and they are consumed periodically. The EAT-*Lancet* diet was disseminated in nine languages and funded by the Wellcome Trust and the EAT forum, a Norwegian-based independent non-profit organization founded by the Stordalen Foundation, the Wellcome Trust, and the Stockholm Resilience Centre [41].

### 3.1. Methodological Limitations of the EAT-Lancet Reference Diet

Despite its novelty, the EAT-*Lancet* reference diet has several methodological limitations. The suggested consumption thresholds for each food group were based mainly on epidemiological data (cohort and cross-sectional studies), with few meta-analyses (one published in 2007). No grading system was employed to rate evidence quality and produce valid recommendations for the selected thresholds. The Commission’s experts made recommendations based on selected data published during the previous decades. For instance, the results of the “Seven countries study” [43], a landmark cross-sectional study initiated in 1958, investigating the relationship between diet and disease, has numerous methodological flaws, and due to its design, cannot provide evidence for the development of informed decisions [44,45], despite being innovative at its time. In the EAT-*Lancet* report, this specific reference is used to introduce the notion that lower meat intake is associated with reduced cardiovascular risk. As Trijsburg noted [13], the EAT-*Lancet* authors based their recommendations on previously published systematic reviews, meta-analyses, and pooled analyses of primary data, added as a supplementary material to the report, without developing a new systematic product to answer each research question. According to Zagmutt [46], the report failed to use systematic methods for the selection of the health impacts of foods and their corresponding risk ratios (RRs), despite the fact that these were crucial to their findings and the proposed diet. The report resembles a narrative review more than a systematic report driving recommendations.

Additionally, the report makes inconsistent use of the scientific literature for associations between food groups and diseases, without any methodology to grade the evidence [47]. Subsequently, attempts to replicate the U.S. mortality calculations proposed by the EAT-*Lancet* report [48] revealed flaws in the methods and assumptions used to estimate the avoided mortalities, calling into question the global conclusions of the Commission. The arbitrary associations of foods with diseases bring to light the concerns raised over nutrition epidemiology [49,50]. For instance, with regard to meat intake, recent state-of-the-art meta-analyses from expert scientists have revealed that the actual magnitude of association between red and processed meat intake and all-cause mortality and adverse cardiometabolic outcomes is very small, but on the other hand, the existing evidence is of low certainty [51]. Similar results were also apparent with regard to cancer mortality and incidence, where the certainty of evidence using the Grading of Recommendations Assessment, Development, and Evaluation (GRADE) [52] approach was low to very low [53]. An akin meta-analysis examining meat intake and cancer outcomes also revealed that low- or very-low-certainty evidence suggests that the adoption of diets with less red/processed meat may induce very small reductions in adverse cancer outcomes [54].

Meta-analyses are performed to gather primary evidence and pool data in order to guide decision making in science, including nutrition and medicine [55]. When old meta-analyses are used to inform decisions, more recent studies are not included, while in parallel, the methodological quality of the meta-analyses is not always up-to-date. For this reason, for each recommendation regarding clinical decisions, either in nutrition or in medicine, the conduction of a systematic review and/or meta-analysis is required to provide up-to-date information for the examined hypothesis, including the most recent evidence. As a result, all clinical practice guidelines and nutrition recommendations must be developed under this methodology, which has been described in detail in the AGREE consortium [56]. In the EAT-*Lancet* report, neither the GRADE [52] nor the AGREE [56] were used to produce evidence tables and guide recommendations regarding dietary targets, although they are considered as the standard in the development of clinical recommendations [55]. Moreover, given that nutrition recommendations have already been criticized deeply for not meeting scientific standards [57,58,59,60], it is important to safeguard sustainable nutrition from this saga. Critical evaluation of the process used to synthesize the evidence when making recommendations for clinical practice (as in the EAT-*Lancet* report) enables users to assess the trustworthiness of these recommendations [61]. Health professionals are increasingly dependent on valid recommendations in everyday clinical practice since they do not always have the time to keep up with the medical literature, and for providing evidence-based practice [61,62]. According to Lunny and associates [61], high-quality systematic review products are required for the identification and pooling of the best available up-to-date evidence in order to inform clinical recommendations. The use of non-systematic methods, as seen in the EAT-*Lancet* report [7], may greatly compromise the validity and reliability of the evidence used to inform the formulation of recommendations—let alone global nutritional policy recommendations—and ultimately leads to over- or under-estimation of the treatment effect estimates and potentially untrustworthy and misleading recommendations [61,63].

According to Kaiser [64], the EAT-*Lancet* report [7] acts as a “brokerage between science and policy”; that is, it failed to meet transparency and replicability standards, providing great statistical uncertainty [48]. For some scientists [65], the narrow manner in describing the measures to tackle broken food systems and lack of health is also questionable. For example, anthropologists [65] noted the ill-suited association between premature death as the main residual of unhealthy dietary choices. Furthermore, they pointed out that the term “healthy diets” was repeated approximately 100 times in the report text. For a solution to be evidence-based, it must be transparent, replicable, and supported by the proper quantification of its impact [46]. Nevertheless, this does not appear to be the case for the report [46], despite the fact that the authors claim to use “the best available evidence” [7].

Apart from methodological limitations, the EAT-*Lancet* reference diet also entails economic limitations and raises concerns regarding the efficacy of the diet to provide nutritional adequacy and the suitability of the diet for specific patient populations.

### 3.2. Economic Limitations of the EAT-Lancet Reference Diet

Low basic income constitutes a major health constraint [66], associated with increased food insecurity [67]. Economic analyses of the reference diet have revealed that the cost of the diet exceeded the household/capita income of at least 1.58 billion people worldwide, making it unaffordable [68,69,70]. Based on 2011 commodities prices, it was calculated that the most affordable version of the EAT–*Lancet* diet cost a global median of USD 2.84 daily (IQR 2.41–3.16), which may be a small fraction of the average income in developed countries, but still, it is an unaffordable amount for poor populations of the world [68]. In rural India, people are currently spending an average of USD 1.00/person/day for their diets and the cost of the EAT-*Lancet* diets ranges between USD 3.00 and 5.00/person/day [71]. Not surprisingly, the higher costs of the reference diet are attributed mainly to fresh fruits and vegetables [68]. Moreover, the cost of the benchmarking diet ranged between 3% and 73% of the national income in several low-income and middle-income countries [69].

Interestingly, although the Commission included high-ranking officials of the World Health Organization (WHO) and FAO, the WHO rushed to drop its sponsorship of the EAT-*Lancet* Commission [72] and withdraw endorsement [73], citing concerns regarding the economic impact of the reference diet on poor, livestock-producing countries. There were concerns that the widespread adoption of the diet could risk jobs and traditional diets linked to cultural heritage [73]. It is believed however, that this move by the WHO to withdraw endorsement was propelled by political interests and motivation. According to Drewnowski [69], the FAO description of sustainable diets is based on four pillars: health, society, economics, and the environment; nevertheless, the suggested reference diet falls short on the social and economic aspects of the definition. Other scientists [46] argued that, at the moment, the reference diet appears to promote a solution favoring high-income countries, without providing solutions for major global health issues, including maternal and child malnutrition.

### 3.3. Nutritional Adequacy of the EAT-Lancet Diet

According to a relevant press release of the UN Italian Representation [74], due to its restrictive manner regarding several food groups, adopting the reference diet might be nutritionally deficient and even dangerous for human health in the long run, particularly for populations with enhanced dietary requirements. Furthermore, the nutritional inadequacy of the diet might enhance the need for dietary supplements or food fortification, which in turn may inflate the cost of the diet. Of note, in this situation, given that the carbon footprint of dietary supplements has never been assessed, the final result of balancing nutrient needs through supplements might, in fact, counterbalance the postulated environmental benefits of the reference diet.

### 3.4. Restriction of Animal-Sourced Foods

Many independent researchers revealed flaws in the analyses behind the EAT-*Lancet* report [46,48,75]. Great concern was also raised regarding the restrictive nature of the reference diet for animal food sources [47]. This restrictive manner concerning animal foods seems to disregard recent meta-analyses published in top journals exonerating the association between meat intake and health [51,54]. Furthermore, as Thorkildsen and Reksnesit [47] pointed out, the report does not consider regional and national differences in the available natural resources. Sustainable production for one country is not always sustainable somewhere else in the world [47], and meat production seems to fall within this category. The report also raised concerns regarding the economic future of countries involved in cattle farming, including Ethiopia and other developing countries. The absolute or relative elimination of foods of animal origin would ultimately end cattle farming and all related activities [74], ending the viability of all the cattle farming companies situated in these countries. However, according to the UN Italian Representation [74], in the EAT-*Lancet* report [7], meat products were “arbitrarily regarded as unhealthy”.

Subsequently, two new members were recently appointed to the EAT-*Lancet* Commission, a Zambian agricultural nutritionist and the General Director’s Representative to Ethiopia at the International Livestock Research Institute, aiming to add heterogeneity to the pool of Commission members and provide advice on this issue [76].

### 3.5. Application of the Reference Diet to Specific Patient Populations

An additional important limitation involves the adoption of the diet by patients with diseases limiting the intake of vegetables and fruits, such as irritable bowel syndrome or inflammatory bowel disease. For these patients, achieving health while following the EAT-*Lancet* diet is unrealistic. Moreover, those with drug-resistant epilepsy following a ketogenic diet are unlikely to adhere to the proposed sustainable dietary regime. According to the WHO, “a standard diet for the whole planet, regardless of the age, sex, metabolism, general state of health and eating habits of each person, has no scientific justification at all” [74].

## 4. Indexes Assessing Dietary Sustainability Based on the EAT-*Lancet* Reference Diet

The publication of the EAT-*Lancet* report [7] initiated the need to “quantify” the sustainability of diets. This resulted in the development of several indexes, many based on the EAT-*Lancet* report [7] and others developed *de novo* (Table 1). In order to fulfill all sustainability criteria, these indexes must also account for the economic, environmental, fair trade, cultural heritage, and population health aspects of sustainability, as previously detailed in Figure 1.

### 4.1. The EAT-Lancet Diet Score

The EAT-*Lancet* diet score [77] was the first by-product of the EAT-*Lancet* report. It is based on 14 food items according to the eight food groups suggested in the reference diet. The suggested energy intake is 2500 kcal/day, as in the reference diet. For each food group, a threshold intake of dry, raw weight is recommended; consumptions below each threshold add one point to the index score (i.e., above the minimum intake or below the maximum intake). Any intake not meeting the threshold of a specific food group receives a score of 0, based on a binary scoring system [77]. With regard to whole grains, roots, and tubers in particular, recommendations were increased to ensure reaching a daily energy intake of 2500 kcal; thus, the suggested intakes were not based on disease prevention or environmental impact suggestions [13].

The use of the reference diet showed beneficial associations with ischemic heart disease and diabetes in the U.K. European Prospective Investigation into Cancer and Nutrition (EPIC)-Oxford cohort [77]. However, an analysis using the U.S. Department of Agriculture (USDA) food database showed that the maximum score can be achieved by consuming a small apple, approximately 200 g of tomatoes, 28 g of nuts, 10 g of extra-fiber all-bran cereals, and nothing else whatsoever each day [80]. For this reason, it has been argued that the results of the score “have no relevance” to the EAT-*Lancet* report [80]; the Commission has not commented on the subject, nor has it endorsed the score.

Cacau et al. [78] spotted several limitations regarding the score. Using reference values in grams does not allow for assessing individual adherence, irrespective of the total energy intake of the participants. For example, the 2500 kcal/d diet cannot apply to infants, young children, or athletes. Furthermore, it does not include intermediate intake and interchangeable group values [78]. An additional bottleneck is that the score does not account for sustainability’s environmental and economic aspects.

Finally, since it is based on the EAT-*Lancet* report, it also carries all the previously mentioned limitations of the reference diet.

### 4.2. The World Index for Sustainability and Health (WISH)

The WISH was designed with the aim to monitor the healthiness and environmental sustainability of the diet of a population [13], based on the EAT-*Lancet* recommendations [7]. However, the authors omitted tubers and starchy vegetables from the scoring, arguing that neither the WHO nor the Global Burden of Disease (GBD) included them in their recommendations. With the removal of the tubers and starchy vegetables food group, the WISH consists of 13 foods and food groups. Its scoring is based on a gradual system according to the classification of each food group as neutral, protective, or negative for human and planet health, without requiring food composition tables or LCA data, as the authors considered that these data are unavailable for different countries [13]. Food groups considered as more protective had a greater consumption recommendation, whereas those deemed as more harmful for human health and the environment were suggested to be consumed in smaller amounts. Moreover, scores are assigned assuming a linear relationship between the component and the health outcomes, with protective food groups providing greater scores with increased consumption and negative food groups providing lower scores when consumed in greater amounts. Additionally, food groups are also divided in three categories based on their environmental impact (low, medium, and high environmental impact food groups).

The index was validated in a small sample of 396 urban Vietnamese men and women using duplicate 24 h dietary recalls [13]. The initial analysis revealed that the score could differentiate between the healthiness and environmental sustainability of the Vietnamese diet.

The index uses reference values in grams, not allowing for the assessment of individual adherence regardless of the total energy intake of the diet [78]. Moreover, it fails to include all intermediate values and interchangeable groups proposed in the EAT-*Lancet* report [78], meaning that for each food group, meeting the threshold or not, is the only parameter affecting the score received, in a binary manner. When half the amount of foods is consumed, no intermediate score can be provided. A total of four distinct sub-scores are added for the calculation of the total WISH, namely, (i) the healthy sub-score, evaluating how healthy the diet is (sum of the eight protective and two neutral food groups), (ii) the less healthy sub-score assessing how unhealthy a diet is (sum of the three “unhealthy” food groups), (iii) the low environmental impact sub-score (sum of the six low environmental impact food groups), and (iv) the high environmental impact sub-score (sum of the three high and four moderate environmental impact food groups) [13]. As a result, one individual may score high in the healthy sub-score but low in the low environmental impact sub-score, indicating a healthy diet for humans, but unhealthy for the planet. Similarly to the EAT-*Lancet* diet score, it inevitably carries all the limitations of the respective reference diet. Finally, although developed as a world index for measuring dietary sustainability, it has only been validated in a Vietnamese sample.

### 4.3. The Planetary Health Diet Index (PHDI)

The PHDI [78] is based on 16 components with proportional scoring, considering the EAT-*Lancet* food groups as energy intake ratios. The components include nuts and peanuts, legumes, fruits, total vegetables, whole grains, eggs, fish and seafood, tubers and potatoes, dairy, vegetable oils, dark green vegetables-to-total vegetable ratio, red and orange to total vegetable ratio, red meats, chicken and substitutes, animal fats, and added sugars. A daily intake of 2500 kcal was set with different intakes from 16 distinct food groups, expressed in two manners, as g/day and as kcal/day [78], according to the EAT-*Lancet* reference diet [7]. The energy contribution of all intake tiers and midpoints proposed for each food group were calculated to the reference diet of 2500 kcal/d. Each component was weighted based on adequacy, moderation, and optimum intake ratio, based on whether intake values suggest a greater or lower adherence to the reference diet assumptions, according to the system indicated by the Dutch Healthy Eating Index (HEI) group [85]. Concerning the scoring system, each of the 16 components of the PHDI provides a maximum of 10 or 5 points, with the total score ranging from 0 to 150 points.

The ELSA-Brasil multicenter cohort validated the index (N = 15,105 men and women aged 35–74 years) [78]. The construct validity and reliability of the index were performed based on the methodology proposed for the HEI [86], according to the relevant Brazilian-revised tool [87]. The internal reliability was evaluated using Cronbach’s α, which had a value of 0.51. For the construct validity, three separate sets of examinations were performed. In the first, linear regression models adjusted for sex and age were used to assess correlations between the PHDI score with selected nutrients. In the second set of examinations, it was assessed whether the PHDI could assess adherence to EAT-*Lancet* recommendations, irrespective of the amount of energy consumed in the diet. In the third, a principal component analysis (PCA) was performed to investigate whether the PHDI had more than one factor explaining the variability of the data. Finally, the degree that the PHDI could discriminate between groups with known differences in the quality of their diets was also evaluated [86]. The results of the construct validity revealed that the PHDI showed a positive correlation with the intake of carbohydrates; PUFA; vegetable proteins; dietary fiber; vitamins A, C, E, and K; folate and thiamine; and several elements, including Fe, K, Zn, Se, Mg, and Cu [78]. Moreover, the PHDI was negatively associated with total and saturated fat, animal protein, cholesterol, MUFA, etc. No association was observed between the index and total protein intake, total energy, Calcium, or Na consumption [78]. The PCA revealed a variety of factors that explain the variability in the PHDI, although none of the 16 components were deemed responsible for a significant proportion of the observed covariance in the data [78].

In parallel, GHGe was calculated to adjust for the ecological aspect of the sustainable diet definition. For this, the “Environmental Footprints of Food and Culinary Preparations Consumed in Brazil” database was used to estimate the carbon footprint of each consumption data, based on the FFQ [88]. Nevertheless, the GHGe was only used to “validate” the index and is not used in the calculation of the score, which is only based on the quantity of the consumed food groups and the respective caloric densities, without any specific weighing according to the GHGe.

A cohort analysis revealed that Brazilians with greater PHDI scores (greater adherence to the EAT-*Lancet* diet) were 24% less likely to be overweight/obese [89]. Moreover, findings from the National Dietary Survey 2017–2018 revealed that the average PHDI score of the Brazilian population reached 45.9 points (95% CI 45.6–46.1), indicating extremely low adherence [90].

An important limitation of the index is that it does not account for the environmental and economic aspects of sustainability.

### 4.4. The Sustainable-HEalthy-Diet (SHED) Index

The SHED Index [79] was based on the EAT-*Lancet* reference diet and the Mediterranean Diet Score (MDS) [83]. It is based on 30 components, namely, healthy eating, dietary consumption, intake of sweetened beverages and bottled water, intake of ultra-processed food and plant-based foods, purchase of organic food, and food consumerism, including food waste and domestic waste streams [79]. In the final index, dietary intake is not accounted for, but sustainable dietary habits are evaluated through several questions (Table 1). The methodology behind the development of the SHED was innovative since it includes a variety of sustainable diet components, without relying solely on dietary intake cutoffs.

The Delphi method was applied to define the exact questions to be included in the questionnaire, with overall agreement on most issues. The participating experts had nutrition, public health, risk-assessment, environmental science, methodology, agriculture, and consumer behavior backgrounds, although no information regarding the number of experts was reported. According to the authors, organic farming was one of the issues not reaching a unanimous expert agreement, in light of the limited agricultural land per person in Israel, thus excluding this particular question [79]. Another issue of concern involved fresh food packaging—given that Israel has a warm climate, and fruits and vegetables are either sold in bulk, or packaged servings. Concerning this issue, the panel agreed not to include information regarding food packaging due to the lack of conclusiveness regarding the benefits and harms between health and the environment [79]. Two questions were assessed on a visual analog scale (VAS) of 100%, namely, compliance with recycling waste and packaging and the proportion of plant-based food in the diet. Regarding dietary intake, a semi-quantitative FFQ [91] with 115 food items, each with nine frequency options, was used to validate food consumption questions. Finally, a PCA was performed to evaluate the loading of each component in the algorithm.

A total of 348 Israeli men and women aged 20–45 years completed the questionnaire. Greater intake of animal protein intake was associated with a lower SHED Index, whereas more recycling efforts were associated with a higher score. As expected, MDS and SHED were highly correlated [79]. Furthermore, the SHED Index was consistent with the EAT-*Lancet* reference diet. No other studies have applied the SHED index to date.

According to the authors, an important limitation of the questionnaire is that it assesses various sustainable diet dimensions without quantifying GHGe, the main metric for evaluating environmental burden [79]. Another limitation is that dietary intake is not calculated or directly accounted for in the index; rather, sustainable dietary habits are evaluated. Furthermore, the index can assess the sustainability of nutritional choices without considering individual economic constraints, falling short of this specific aspect of the sustainable diet definition [11].

### 4.5. Indice de Dieta Saludable y Sostenible (IDSS)

The Mexican IDSS [80] was developed according to the EAT-*Lancet* reference diet. It is constructed of 13 food groups, including whole-grain foods, tubers and starchy vegetables, vegetables, fruits, milk and by-products, beef or pork, chicken and other birds, eggs, fish and seafood, legumes/soybeans/tree nuts, saturated fats, unsaturated oils, and added sugars. PCA was used to assess the loading of each component. The scoring is categorized in ≤5, 6, 7, 8, or ≥9 total points.

The index was validated in a sample of Mexican adults (N = 11,506) from the National Health and Nutrition Survey 2018–2019. The results revealed that men with higher scores demonstrated a lower prevalence of obesity, although the association between IDSS and obesity was not significant in women [80].

## 5. Other Indexes Assessing Dietary Sustainability, Developed Independently of the EAT-*Lancet* Reference Diet

### 5.1. The Healthy and Sustainable Diet Index (HSDI)

The HSDI [81] was the first reported effort to devise an index assessing dietary sustainability. The proposed methodology suggested its validation in a sample of 247 Australian young adults, aged between 18 and 30 years, participants of the Connecting Health and Technology study. According to the published protocol, 4 days of food and beverage images would be analyzed from a mobile food record (mFR) application. The mFR would calculate servings of eggs, red meat, dairy, fish and poultry, fruit and vegetables (including seasonality), ultra-processed energy-dense nutrient-poor foods and beverages, individually packaged foods, and plate waste [81]. This would result in the development of a prediction model for the HSDI. However, despite its novelty, the final product was never published.

### 5.2. The Sustainable Diet Index (SDI)

The SDI was designed to assess dietary sustainability, incorporating individual multidimensional indicators of sustainability [82], based on the FAO’s definition of sustainable diets [11]. It includes seven indicators categorized into four standardized domains, representing the diet’s environmental, nutritional, economic, and socio-cultural aspects.

An annual organic, previously validated, semi-quantitative 264-item FFQ [92] was used to collect data regarding the dietary intake of participants. The reported consumption and the probability of adequate nutrient intake (PANDiet) [84] were calculated for each participant, assessing dietary adequacy. Apart from nutrient adequacy, an additional sub-index was developed, assessing the adequacy of energy intake to meet energy needs.

Concerning the environmental impact, a specifically developed database of environmental indicators of raw agricultural products was used, assessing three indicators: GHGe, primary energy consumption, and land occupation. A partial score was calculated for each food product. The total dietary and environmental impact was estimated by multiplying the score by the quantity of food consumed, accounting for the agricultural production methods in each case.

The share of organic food in the diet was included in the index (biodiversity preservation), and the individual daily monetary cost of each diet was computed by multiplying the quantity of consumed goods by their price. Furthermore, an index was developed, evaluating the diversity of purchase places other than supermarkets and an additional one assessing the intake of ready-made products.

Each of the four domains (environmental, nutritional, economic, and socio-cultural aspects of the diet) receives a score between 1 and 5; thus, the total score can range from 4 to 20, with greater scores indicative of more sustainable diets.

The index was validated in a sample of 29,388 participants in the Nutri Net-Santé cohort study [82]. The results revealed that participants exhibiting a high SDI were concordant with the proposed sustainable diets in the literature.

As the authors noted [82], selecting a 1–5 rating scheme in each domain has an important effect on the index development. The authors opted for five categories, one for each sustainable diet indicator, aiming to discriminate participants without having many categories. Additionally, equal weights were given to the four sub-indexes, reflecting the absence of hierarchy in the FAO’s sustainable diet definition [11]. Additionally, the authors expressed their interest in expanding the index in the future, aiming to include water footprint, fair trade, or crop treatment frequency indexes [82]. Last, but not least, since the index is based on the French recommendations for dietary intake, adaptation of the score and extrapolation outside France are not warranted.

Publication of the SDI gave the idea for cultural adaptations of the index in other countries. In this context, a Malaysian SDI was proposed and is currently being developed [81].

## 6. Conclusions

Despite the various tools and indexes developed to assess dietary sustainability, a gold standard is still missing. This is because the sustainable diet option is yet novel, requiring further research to understand the concept and reach a consensus on its aspects. Furthermore, most of the indexes are based on the EAT-*Lancet* reference diet, which has raised concerns regarding its limitations. After all, indexes developed using an a priori method tend to inherit various methodological limits [93]. However, no other alternative reference diets have been developed, and this indicates that research should be shifted towards new prototype diets before the development of more indexes. Undoubtedly, the EAT-*Lancet* report was the first attempt to solve a difficult problem and it was not within the scope of the present review to offer criticism without aiming to improve the development of future reference diets and upgrade the science of nutrition. Version 2.0 of the EAT-*Lancet* report is expected to be published in the year 2024, acknowledging that a consensus regarding the global targets is still missing [42]. Clearly, a takeaway point from the present review is that the design of one diet that is appropriate for all cultures, populations, patients, and geographic locations—that is environmentally friendly at the same time—is a difficult, if not impossible task.

For this, the development of sustainable and healthy diet recommendations that are region-specific and culturally specific, while at the same time, encompass all aspects of sustainability, is required. Researchers argue that increasing population adherence to the existing government dietary guidelines in each country would be a more realistic approach to improving the health and environmental impact of the consumed diets [94]. According to Springmann [95] and Kovacs [96], however, the current food-based dietary guidelines are incompatible with climate change, freshwater, land use, and nitrogen targets, and this should be corrected. As a result, the FAO and several individual researchers have pledged the incorporation of sustainability in the national and global dietary guidelines [95,97,98]. According to the Intergovernmental Panel on Climate Change, every country needs to evaluate how land and natural resources can be used for food production in the most sustainable manner, and consider socio-economic, natural site-specific, and cultural particularities of each geographic area before making suggestions for a sustainable diet policy [99].

The principles of sustainable healthy diets are set to provide flexible roadmaps for policy actors [100]. On the other hand, for a sustainable and healthy diet to be quantified, the dimensions selected for each index require meticulous assessment by relevant indicators [101]. According to Eme [102], and as demonstrated in the present review, the evidence basis for selecting specific and robust indicators for sustainable diet indexes is frequently weak, fragmented, and arbitrary. Furthermore, great heterogeneity is apparent among the included indicators and their weight on population health [100]. Many indexes do not adequately account for the diets’ environmental impact, whereas others fall short in the economic impact domain. In parallel, consideration of the water footprint is missing from most indexes.

Last, but not least, the ethical aspect of the sustainable diet is under-examined in the currently available indexes. Fair trade is only accounted for in the SHED index, but the remaining authors have failed to incorporate this component in their sustainability scores. Environmental ethics are linked to food choice morality, and meeting social standards can often be more costly than meeting nutrient requirements [69,103].

In summary, it appears that the methodology behind the development of indexes to assess dietary sustainability is demanding, requiring the consideration of several sustainability aspects. Furthermore, the need for reliable, evidence-based prototype diets is also a demanding task, in order to ensure the trust of the public and the scientific community. 

## Figures and Tables

**Table 1:** Indexes assessing dietary sustainability.

Index Name	Origin	Main Domains of the Index	Components	Dietary Intake Domains (Foods and Food Groups)	Environmental Impact	Based on the EAT-*Lancet* Reference Diet [7]	Score Range
EAT-*Lancet* diet score [77]	U.K.	Dietary intake	14 food items, each providing a score of 0 or 1.	Whole grains, tubers, starchy vegetables (potatoes and cassava), vegetables, fruits, dairy foods, protein sources (meat, eggs, fish, legumes, nuts), added fats, added sugars.	Not accounted for	Yes	0–14
WISH [13]	Global (The Netherlands, Italy, Brazil, USA)	Dietary intake	13 components, each scored between 0 and 10.	Whole grains, vegetables, fruits, dairy foods, red meat, fish, eggs, chicken/poultry, legumes, nuts, unsaturated fats, saturated fats, added sugars.	GHGe, land use, eutrophication, acidification, scarcity weighted water	Yes	0–130
PHDI [78]	Brazil	Dietary intake	16 food items, each providing a maximum score of 10 or 5 and a minimum of 0.	Nuts and peanuts, legumes, fruits, total vegetables, whole grains, eggs, fish and seafood, tubers and potatoes, dairy, vegetable oils, dark green vegetables/total vegetable ratio, red and orange/total vegetable ratio, red meats, chicken and substitutes, animal fats, added sugars.	Not accounted for	Yes	0–150
SHED Index [79]	Israel	Healthy eating (overall dietary consumption), drinking habits (intake of sweetened beverages and bottled water), sustainable eating (plant-based), socio-cultural aspects (organic foods, food consumerism), intake of ultra-processed and plant-based foods, environmental aspects (food waste, domestic waste streams)	30 items, each with a different weight to the score.	The healthy eating domain includes consumption frequency questions regarding meat products, plant-based foods, fruit/vegetable variety, preference for plant-based over animal products, drinking water preference, low-salt products, ultra-processed products, low-sugar foods, sweetened beverages, sweets, salt intake, recycle food scraps with a composter, preferring foods made in the country.	Accounted for, although it does not quantify GHGe	Yes, and the MDS	0–100
IDSS [80]	Mexico	Dietary intake	13 food items, each providing a binary score of 0 or 1.	Whole-grain foods, tubers and starchy vegetables, vegetables, fruits, milk and by-products, beef/pork, chicken and other birds, eggs, fish and seafood, legumes/soybeans/tree nuts, saturated fats, unsaturated oils, added sugars.	Not accounted for	Yes	0–13
HSDI [81]	Australia	NR	NR	NR	NR	No	NR
SDI [82]	France	Environmental, nutritional, economic, and socio-cultural aspects of the diet	4 components, providing a score of 1–5.	A nutritional sub-index reflects the adequacy between energy intake and needs. The PANDiet is included as a sub-index, assessing the adequacy in nutrient intake based on the French recommendations for 24 nutrients.	Accounted for	No	4–20

GHGe, greenhouse gas emissions; HSDI, Healthy and Sustainable Diet Index [81]; IDSS, Indice de dietasaludable y sostenible [80]; MDS, Mediterranean diet score [83]; PANDiet, probability of adequate nutrient intake [84]; PHDI, Planetary Health Diet Index [78]; SDI, sustainable diet index; SHED, Sustainable-HEalthy-Diet [79]; U.K., United Kingdom; USA, United States of America; WISH, World Index for Sustainability and Health [13].

## Data Availability

Not applicable.
